# Trends in Susceptibility to Aggressive Periodontal Disease

**DOI:** 10.16966/2378-7090.197

**Published:** 2016-04-25

**Authors:** Nishat Shahabuddin, Kathleen Boesze-Battaglia, Edward T Lally

**Affiliations:** 1 Department of Biochemistry, School of Dental Medicine, University of Pennsylvania, Philadelphia, USA; 2 Departments of Pathology, School of Dental Medicine, University of Pennsylvania, Philadelphia, USA

**Keywords:** Aggressive periodontitis, Juvenile periodontitis, *Aggregatibacter Actinomycetemcomitans*, LtxA

## Abstract

*Aggregatibacter actinomycetemcomitans* is a gram-negative microbe involved in periodontitis. Strains with varying degrees of virulence have been identified, in healthy and periodontally compromised individuals alike. Hosts mount differential immune responses to its various serotypes and virulence factors. Studies have explored host immune response in terms of antibody titers, leukocyte responses, and specific inflammatory mediators, questioning the ways in which the infectious microorganism survives. This mini-review will identify the key themes in immune response patterns of individuals both affected by and free from aggressive periodontal disease, thereby using it to understand various forms of periodontitis.

## Background

### Periodontitis: An interplay between genetics and the microbial environment

Protective inflammation manifests as attachment loss in periodontitis in select individuals. The 2012 Polymicrobial Synergy and Dysbiosis (PSD) model of periodontal disease proposes that pathogens have distinct roles in a diverse biofilm such that there is specific, localized destruction in certain areas of the oral cavity [1]. Thus, the diversity of the biofilm may result in specific, localized destruction in some patients if only certain areas of the film are particularly pathogenic. The PSD model focuses on the importance of *Porphyromonas gingivalis* as a keystone pathogen; however, other bacteria have been acknowledged by the American Academy of Periodontology as having an important role in the development of periodontal disease. *Porphyromonas gingivalis, Aggregatibacter actinomycetemcomitans*, and *Tannerella forsythia* are three consensus periodontal pathogens implicated in periodontal diseases.

The PSD model of disease also attributes periodontitis to host defense mechanisms. Protective mechanisms achieve a homeostatic balance with the microenvironment to varying degrees in individuals. Innate and humoral immune mechanisms may be hyper-responsive to, or perhaps deficient towards, a particular stimulus, presumably for genetic reasons.

### Current classifications of periodontitis

The different types of periodontitis are classified by neither the associated bacteria, nor the molecular basis of host susceptibility to different periodontal diseases due to limitations in understanding the disease process. Historical classifications focused on the time of onset and rate of progression of disease [2,3]. In 1999, the American Academy of Periodontology (AAP) developed the most recent classification of periodontal diseases to distinguish between Aggressive Periodontitis (AgP) and Chronic Periodontitis (CP). Aggressive Periodontitis (AgP) patients present with rapid destruction and bone loss accompanied by minimal inflammation at the time of diagnosis, often after irreversible damage has occurred. Chronic Periodontitis (CP), by contrast, is usually seen in patients above 35years of age, frequently with a buildup of dental plaque that is suggestive of poor oral hygiene. The body mounts a strong, pro-inflammatory response to the several pathogenic organisms present in sub gingival plaque. It progresses at a slow rate relative to AgP. Both are found in localized and generalized forms, based on the number of affected sites, but they vary in rate of progression [4]. Diagnoses of both infectious diseases can be made clinically, through measurement of probing depths, and radiographically, through analysis of bone levels.

### Previous classifications of periodontitis

Classifications of periodontitis prior to 1999 included patient age as a parameter of diagnosis, distinguishing “early-onset” periodontitis from “adult” periodontitis. Early-onset periodontitis was subdivided into pre-pubertal, juvenile, and rapidly progressive forms. Pre-pubertal and juvenile forms appeared to be associated with specific bacteria; the aforementioned *A. actinomycetemcomitans* was the putative pathogen in Localized Aggressive Juvenile Periodontitis (LJP), a disease which affected adolescents by causing rapid, localized destruction at the central incisors and first molars (Table 1) [5].

There were several challenges to this classification. To illustrate by example, a young adult, 25 years of age, may exhibit classic symptoms of LJP: localized destruction limited to the central incisors and molars. One might believe that the destruction progressed aggressively based on the young age of the patient. However, without a history of onset of periodontitis, it is difficult to claim that the patient exhibits LJP, or, more accurately, a history thereof [4]. It may be discerned from such an example that the terms “early-onset” and “adult periodontitis” were arbitrary in delineating boundaries. To expand, consider an alternative instance of a 16-year-old patient displaying symptoms of Adult Periodontitis, classically associated with inflammation and poor oral hygiene, rather than the acute destruction associated with Early-Onset Periodontitis. It would seem inappropriate to diagnose a juvenile as having “adult” periodontitis. Thus, the benefit of the 1999 Classification is that the distinction between Aggressive/Chronic forms rather than Juvenile/Adult forms circumvents ambiguous variables: age boundaries and age of onset.

However, the new nomenclature decreases granularity in classifications of periodontitis, which is unfortunate given that certain forms of periodontitis may be better distinguished etiologically with higher levels of granularity. However, etiologically, this granularity may be important, especially if certain forms of periodontitis are associated with specific microorganisms. If *A. actinomycetemcomitans* is an etiologic agent of LJP, as literature suggests [6], then it may be ideal to classify LJP as a disease of its own and not subcategorize it under AgP. The new nomenclature thus closely but perhaps artificially groups LJP/LAP with different forms of periodontitis such as GAgP. One may argue that the generalized form of AgP is due to a LAP *A. actinomycetemcomitans* infection having spread, or a certain genetic defect in responding appropriately to *A. actinomycetemcomitans*. Alternatively, one may argue that GAgP is a disease that is etiologically unrelated to LAP. Thus, in terms of understanding the etiology of periodontal diseases, it is debatable as to which classification is most accurate and helpful. The focus of this review is differential immune response susceptibility in the host that could facilitate microbial infection, particularly in response to *A. actinomycetemcomitans* as an etiologic agent of AgP, that may help clarify classifications of periodontitis.

### Origin/Epidemiology of the JP2 infection in LAP

The JP2 strain is a subset of serotype b of *A. actinomycetemcomitans*, and it contains strong associations with LAP [7]. Those with the JP2 strain of *A. actinomycetemcomitans* have a high relative risk for developing aggressive periodontitis [8]. The virulent subset is thought to have emerged approximately 2,000 years ago in North Africa [9]. Interestingly, the JP2 clone in particular does not appear to have spread to non-African populations despite the widespread migration of the individuals of African origin [9]. It is not known whether there is some host susceptibility that renders certain populations vulnerable to the disease. The limited spread of the LAP facilitates its use as a model for understanding periodontal disease.

### Highly leukotoxic clones

A 530-base pair (bp) deletion in the JP2 genome results in secretion of large amounts of leukotoxin (LtxA) which likely contributes to increased virulence [10]; JP2 clones are referred to as “highly leukotoxic” [11]. The toxin induces apoptosis in mononuclear leukocytes (MNLs), but the molecular mechanism of cytotoxicity has yet to be elucidated. Secretion of LtxA into the GCF may allow *A. actinomycetemcomitans* to lyse lymphocytes in the microenvironment and effectively delay an immune response to the oral biofilm. Thus, the microbe would have ample time to proliferate within the host [12]. Like the rare nature of LAP, the 530- bp is useful as a marker for tracing the disease and understanding one particular mechanism of periodontitis.

## State of Affairs

### Humoral immune response susceptibility in periodontitis

It is not clear whether humoral immune responses to *A. actinomycetemcomitans* terminate the spread of infection or, alternatively, lead to a hyper-responsiveness. Furthermore, failure to mount a humoral response may indicate genetic susceptibility which facilitates microbial infection [13]. Development of a specific response may reveal information about the timing and mechanism of *A. actinomycetemcomitans* associated tissue destruction, ultimately highlighting when *A. actinomycetemcomitans* infection is most preventable. However, there is inconsistent correlation of the immune response with clinical presentation of symptoms [14,15].

The humoral response to *A. actinomycetemcomitans* appears to be protective, rather than a hyper-inflammatory means of periodontal destruction. Vlachojannis et al. 2010 [16] find that that development of IgG antibodies to various *A. actinomycetemcomitans* serotypes is associated with clinical diagnosis of periodontal status. The National Health and Nutrition Examination Survey (NHANES) were analyzed for serum antibodies to various periodontal pathogens. Edentulous patients generally displayed lower antibody titers to pathogenic organisms such as *A. actinomycetemcomitans* and other “red complex” species such as *P. gingivalis*. Presumably, the failure to mount a humoral response is associated with clinical presentation of symptoms. It should be noted that although the Vlachojannis study is specific to *A. actinomycetemcomitans*, it distinguishes neither between age cohorts nor between AgP and CP.

A study by Casarin et al. 2010 [17] finds that adult GAgP patients display lower IgG levels towards *A. actinomycetemcomitans* and *P. gingivalisin* comparison with adult patients with GCP. Generalized forms of periodontitis that are rapidly progressive and aggressive may be etiologically related to forms of periodontitis that involve failure to mount an immune response; Thus Casarin’s findings echo those of the NHANES study, which suggested that humoral immune response susceptibility to multiple organisms existed in individuals with generalized destruction.

In addition, a LAP-specific study from Mette Rylev in 2011 [18] showed that individuals with JP2 infections would uniquely react to certain *A. actinomycetemcomitans* antigens. The study found a uniquely strong humoral response to LtxA in a Moroccan cohort [8].Thus, LAP as a model for periodontal disease may serve as a useful study tool for understanding other types of periodontitis, given that all diseases potentially involve a weak humoral immune response.

### Innate immune response susceptibility in periodontitis

Innate immune mechanisms also differ between patients with periodontitis and periodontally healthy patients [19]. In an LAP-specific study, Fine et al. 2013 [20] finds low levels of antimicrobial Lactoferrin-iron (Lf-iron), minimal *A. actinomycetemcomitans* agglutinating activity, and high killing activity against gram-positive bacteria, potentially reducing competition for *A. actinomycetemcomitans*. Fine et al. [20] posits that lower levels of Lf-iron facilitate *A. actinomycetemcomitans* colonization [21]. A lack of functional IgA, an agglutinating agent, may fail to facilitate *A. actinomycetemcomitans* aggregation and subsequent elimination.

**Figure 1:**
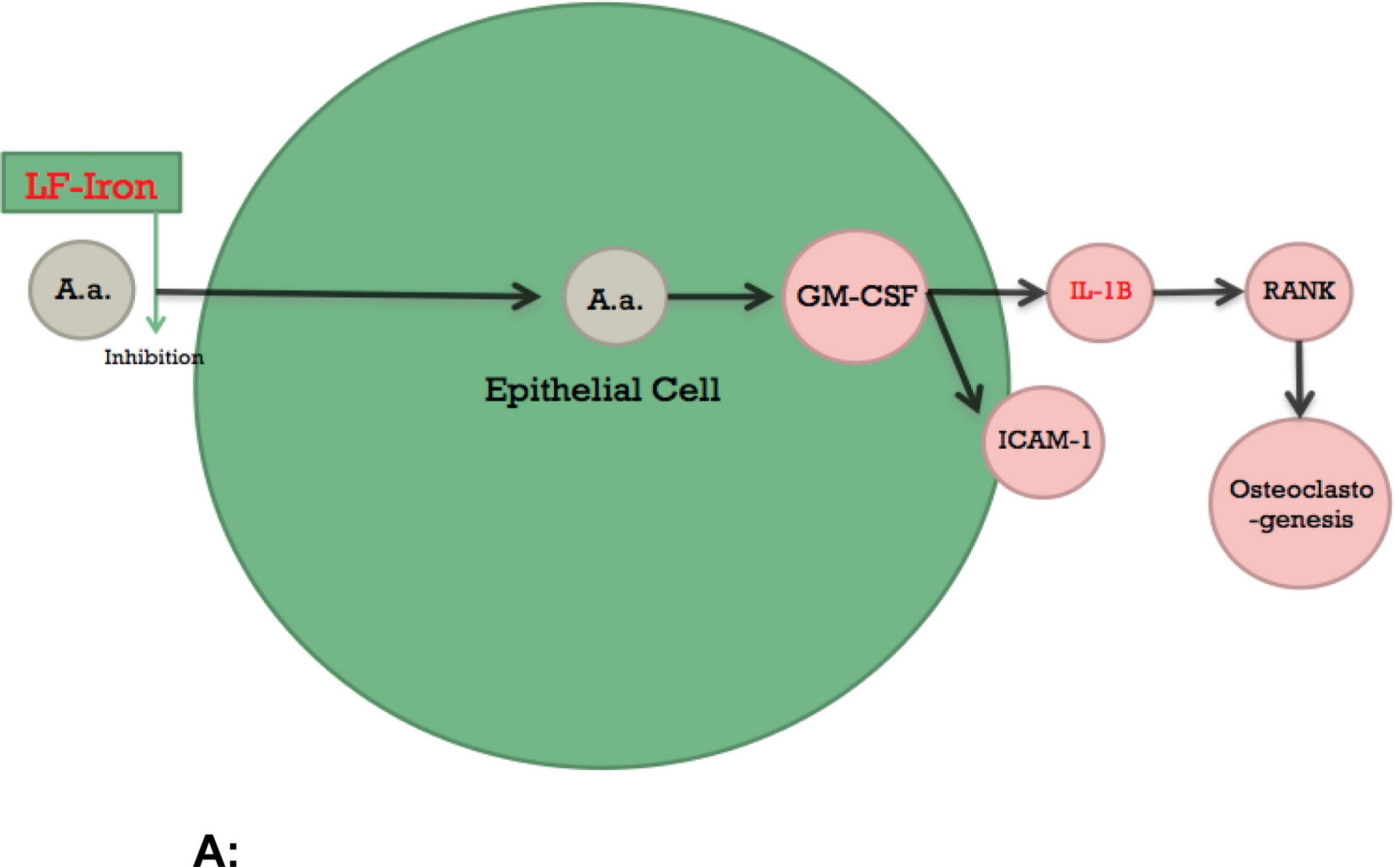

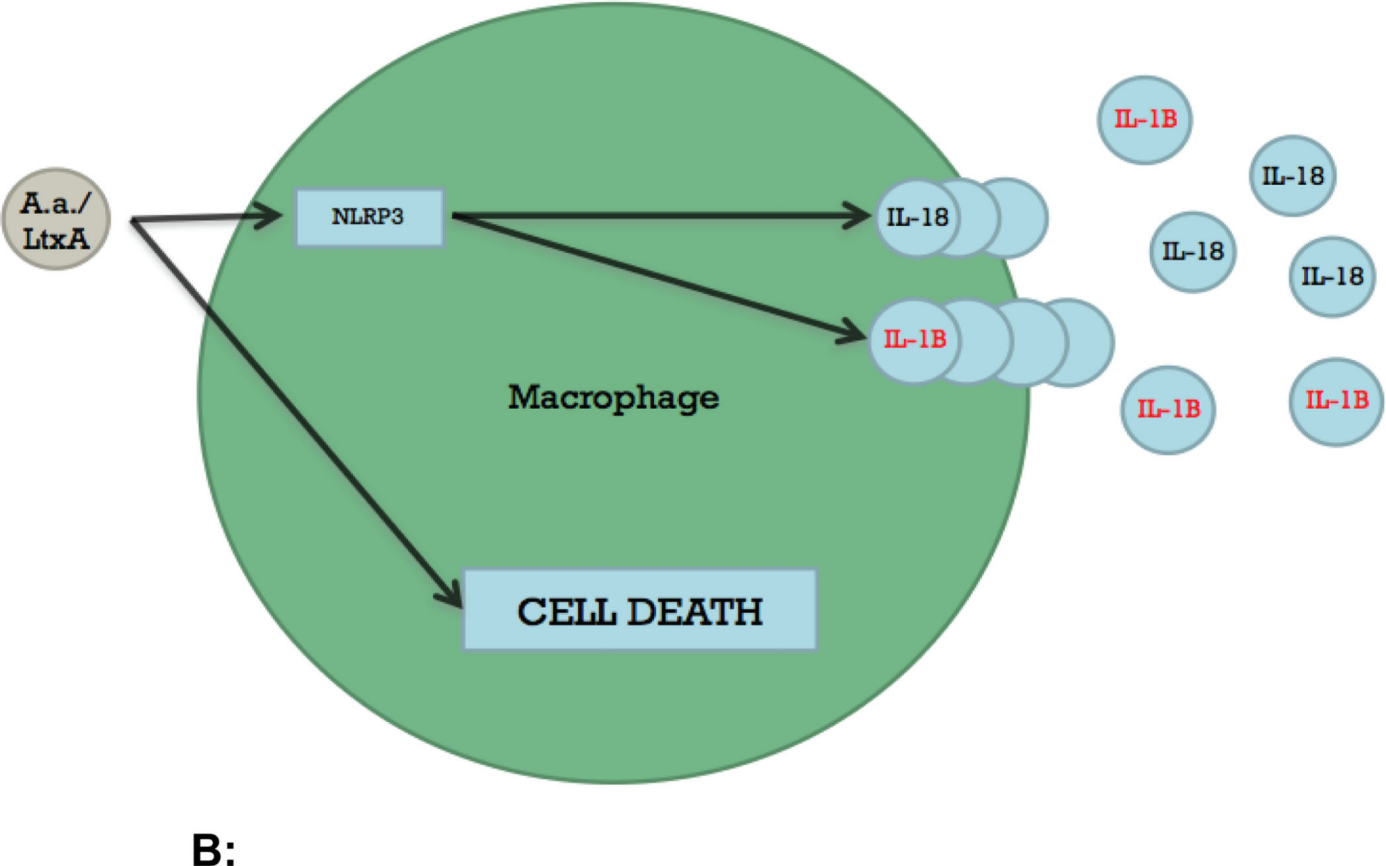

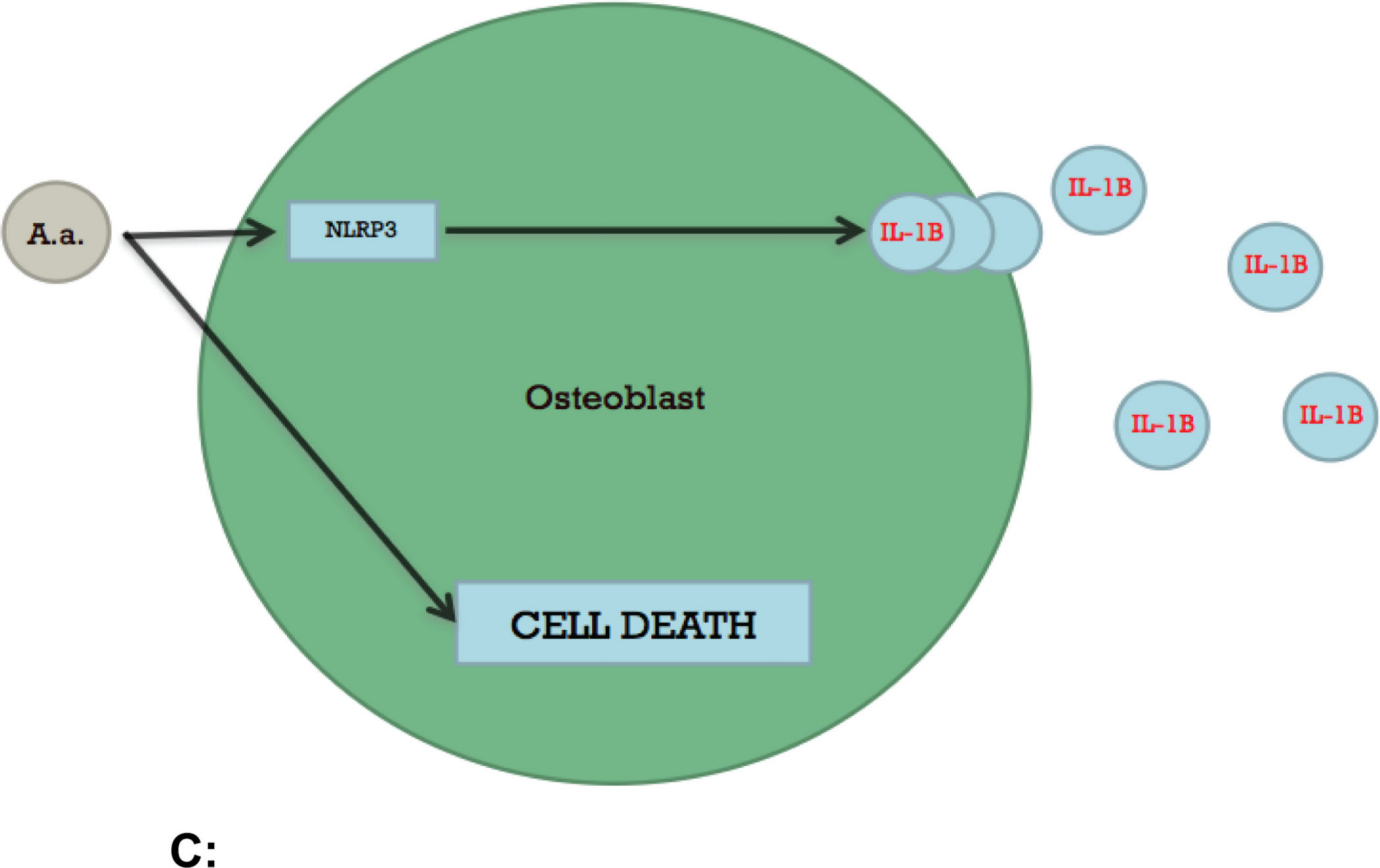

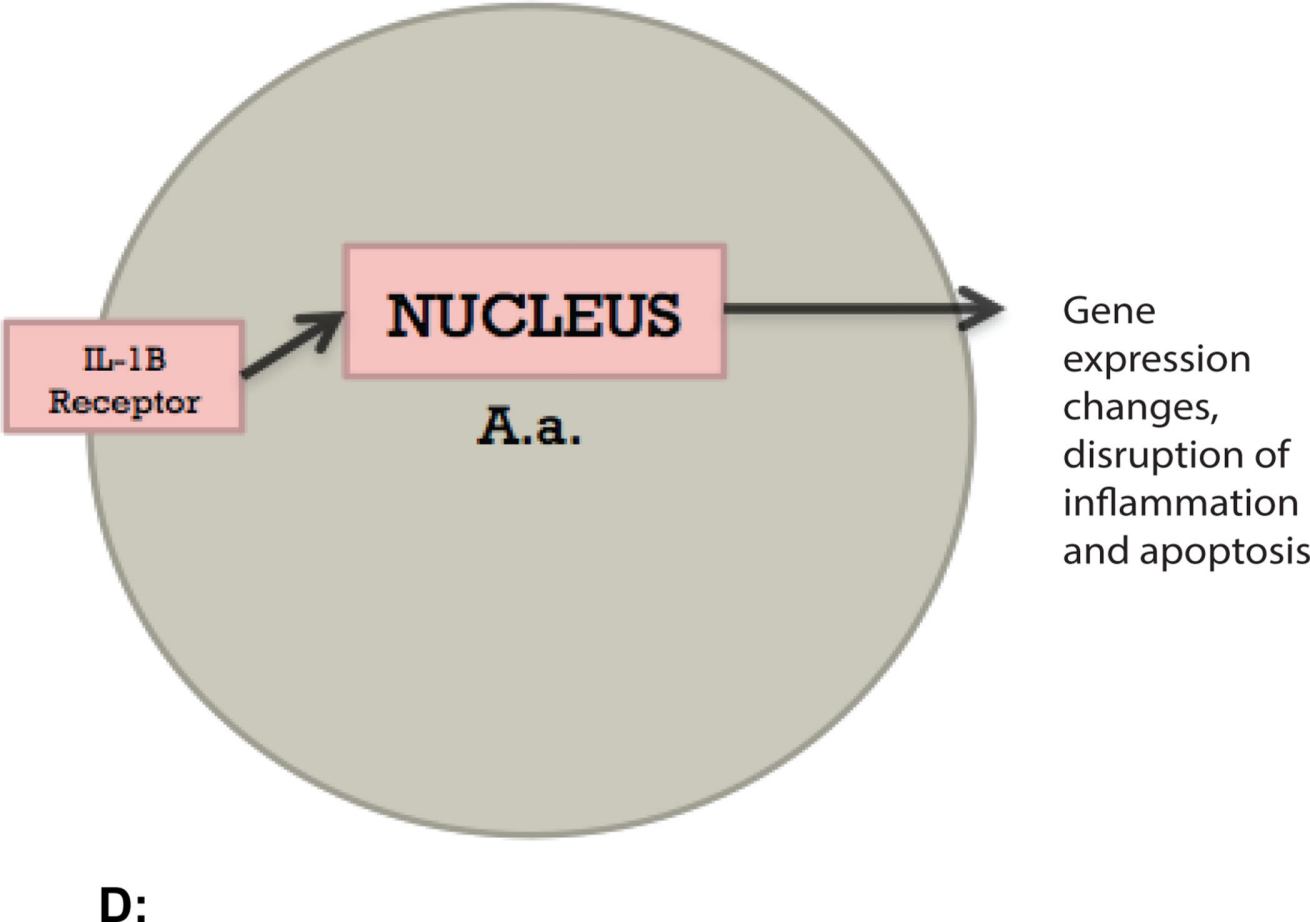
**A:** In epithelial cells, *A. actinomycetemcomitans* leads to in vitro upregulation of GM-CSF and ICAM-1. IL-1B has a role in osteoclastogenesis. Lactoferrin-Iron has antimicrobial effects **B:** In macrophages, *A. actinomycetemcomitans* leads to upregulation of the NLRP3 inflammasome complex, cell death, and increased secretion of IL-18 and IL-1B **C:** In osteoblasts, *A. actinomycetemcomitans* leads to upregulation of the NLRP3 inflammasome complex, cell death, and increased IL-1B secretion **D:** The IL-1B receptor in *A. actinomycetemcomitans* localizes to the nucleus and affects expression of virulence factors

The innate immune system also involves Intercellular Adhesion Molecule-1 (ICAM-1). This pro-inflammatory cell surface molecule is involved in extravasation of lymphocytes, osteoclast formation, and interactions between leukocytes. It is the receptor for Lymphocyte function-associated antigen-1 (LFA-1, CD11a/CD18b), an integrin with which LtxA interacts [12,22]. Umeda et al. [23] find that AgP and CP patients up regulate intercellular adhesion molecule-1 (ICAM-1) and granulocyte macrophage-colony stimulating factor (GM-CSF) in comparison to healthy controls (Figure 1A). In vitro studies with epithelial cell lines showed up regulation of pro-inflammatory genes such as ICAM- 1 in response to *A. actinomycetemcomitans*. Other oral pathogens are not reported to have such effects [24].

Umeda further highlights that pathways downstream of GM-CSF and ICAM-1 expression involves cytokines such as Receptor activator of nuclear factor kappa-B (RANK), which are involved in osteoclastogenesis (Figure 1A). Osteoclasts, which resorb bone, are key in periodontal bone loss [22,25–27]. Although levels of the cytokines may be elevated due to other organisms; the data suggests there may be an over expression of pro-inflammatory mediators in both AgP and CP, suggesting a mechanistic link between the two forms of periodontitis. While studies from Fine et al. [20] suggest a protective role for the innate immune system in periodontitis, studies from Umeda et al. [23] suggest that the innate immune system is hyper-responsive in periodontitis [20,23]. Studies related to LAP and A. *actinomycetemcomitans* have not clarified the role of the innate immune system in periodontitis.

### Candidates for immune response susceptibility

Individuals affected by LAP have immune responses that differ from those in healthy individuals [28,29]. In vitro studies serve as platforms for exploring what the specific differences may be. Kelk et al. [30] found that LtxA resulted inIL-1B secretion, IL-18secretion, and cell death in macrophages (Figure 1B) [30,31]. In addition, incubation with *A. actinomycetemcomitans* leads to an increase in NLRP3 inflammasome gene expression, a complex associated with pathogen-associated molecular patterns (PAMPs), and inflammatory pathways.

The significance of IL-1B extends to osteoblasts as well. These bone-forming cells deposit the mineralized matrix that is degraded in individuals with periodontal bone recession. Zhao et al. [32] found that a human osteosarcoma cell line responded to *A. actinomycetemcomitans* incubation in a manner that led to cell death. Increased transcription and translation of NLRP3 and associated adaptor molecules was observed, eventually resulting in cell death and IL-1B secretion (Figure 1C) [33,34]. *A. actinomycetemcomitans*-induced osteoblast cell death may increase the severity of periodontitis.

Paino and colleagues conducted several studies to explore the consequences of high IL-1B levels within the GCF. Data suggests that *A. actinomycetemcomitans* contains an IL-1B receptor that localizes to proteins involved in gene expression (Figure 1D) [35,36].Candidates for affected genes include adhesins and biofilm-forming proteins which enhance *A. actinomycetemcomitans* virulence [37].

Thus, if inflammasomes and IL-1B are differentially expressed in periodontitis, there may be evidence for a hyper-responsive trait that leaves individuals prone to not only LAP but also CP and GAgP. Given that LAP is associated with minimal inflammation; the pro-inflammatory pathways may be misregulated in a unique, highly localized fashion that may help to clarify how some of these markers are involved in disease.

## Conclusions and Future Directions

Both differential microbial virulence and differential host susceptibilities contribute what is ideally a commensal relationship in periodontal health [1]. Microbes may possess competitive advantages that allow them to colonize the oral cavity with ease. Alternatively, a host defect may lead to the same end result. Such colonization may soon be followed by a hyper-inflammatory reaction in the host or perhaps a failure to respond. There are gaps in the literature regarding the factors that modulate host-pathogen relationship in periodontitis, making nomenclature and categorizations of a periodontal disease a challenge. Different classifications of periodontitis may exhibit patterns in the process of periodontal destruction, and may not be highly pathologically distinct. The nomenclature that recognizes pathological or mechanistic differences in the types of periodontitis may be doing so artificially. Conversely, forms of periodontitis may be unique in their pathology, in which case grouping together different infections risks misdiagnosis of disease.

In order to attempt to understand the progression of periodontal disease and improve diagnoses, the studies highlighted in this review provide evidence for patterns related to *A. actinomycetemcomitans* as an etiological agent of LAP. While studies of the humoral immune response generally suggest that there exist a defect in protective mechanisms in periodontitis, studies of the innate immune response do not clearly demonstrate whether it is protective or hyper-responsive. Further studies of IL-1B pathways, inflammasome pathways, and salivary antimicrobial molecules may lead to the identification of a deficiency common to several types of periodontitis.

Understanding the nature of host susceptibility through studies of *A. actinomycetemcomitans* in LAP will clarify distinctions or similarities between different classifications of periodontitis, further delineating conditions in which microbial colonization is particularly relevant. Future studies may consider exploring how expression of such presumed susceptibilities change over time, perhaps once a disease is terminated. If patterns exist in that regard, then perhaps there exists yet another way to understand the nature of periodontal diseases.

## Figures and Tables

**Table 1:** A simplified outline of the types of periodontitis

General characteristics	1989 Classification			1999 Classification	
Rapid rate of destruction, Seen in families, Seen in younger individuals, Accompanied by minimal inflammation	EARLY ONSET			AGGRESSIVE	
	Pre-pubertal	Juvenile Frequently LJP)	Rapidly Progressive	Localized (LAP)	Generalized (GAgP)
	Localized	Generalized			
Slower rate of destruction, Seen in individuals with poor oral hygiene, Seen in older individuals, Accompanied by inflammation	ADULT (AP)			CHRONIC (CP)	
	Localized	Generalized		Localized	Generalized (GCP)
